# A single vaccination of commercial broilers does not reduce transmission of H5N1 highly pathogenic avian influenza

**DOI:** 10.1186/1297-9716-42-74

**Published:** 2011-06-02

**Authors:** Okti Poetri, Annemarie Bouma, Ivo Claassen, Guus Koch, Retno Soejoedono, Arjan Stegeman, Michiel van Boven

**Affiliations:** 1 Faculty of Veterinary Medicine, Department of Infectious Diseases and Public Health, Bogor Agricultural University, Bogor, Java, Indonesia; 2 Faculty of Veterinary Medicine, Department of Farm Animal Health, Utrecht, The Netherlands; 3 Central Veterinary Institute (Wageningen University and Research Centre), Lelystad, The Netherlands; 4 Centre for Infectious Disease Control, National Institute for Public Health and the Environment, Bilthoven, The Netherlands

## Abstract

Vaccination of chickens has become routine practice in Asian countries in which H5N1 highly pathogenic avian influenza (HPAI) is endemically present. This mainly applies to layer and breeder flocks, but broilers are usually left unvaccinated. Here we investigate whether vaccination is able to reduce HPAI H5N1 virus transmission among broiler chickens. Four sets of experiments were carried out, each consisting of 22 replicate trials containing a pair of birds. Experiments 1-3 were carried out with four-week-old birds that were unvaccinated, and vaccinated at day 1 or at day 10 of age. Experiment 4 was carried out with unvaccinated day-old broiler chicks. One chicken in each trial was inoculated with H5N1 HPAI virus. One chicken in each trial was inoculated with virus. The course of the infection chain was monitored by serological analysis, and by virus isolation performed on tracheal and cloacal swabs. The analyses were based on a stochastic SEIR model using a Bayesian inferential framework. When inoculation was carried out at the 28^th ^day of life, transmission was efficient in unvaccinated birds, and in birds vaccinated at first or tenth day of life. In these experiments estimates of the latent period (~1.0 day), infectious period (~3.3 days), and transmission rate parameter (~1.4 per day) were similar, as were estimates of the reproduction number (~4) and generation interval (~1.4 day). Transmission was significantly less efficient in unvaccinated chickens when inoculation was carried out on the first day of life. These results show that vaccination of broiler chickens does not reduce transmission, and suggest that this may be due to the interference of maternal immunity.

## Introduction

Infection of poultry with highly pathogenic avian influenza (HPAI) virus strains invariably results in high mortality rates and substantial economic losses [1-6]. It is now almost 15 years ago that the first outbreaks of highly pathogenic H5N1 viruses were reported in South East Asia. Since then, the disease has become endemic in some countries [7]. Outbreaks caused by infection with H5N1 viruses resulted in the death of millions of birds either from the disease, or by culling. In addition, hundreds of human infections, including 306 fatal ones, have been reported [8].

Upon detection of an outbreak in commercial poultry, a set of control measures, including culling of infected flocks, is implemented [2,9,10]. Eradication of the H5N1 virus from poultry has been successful in some countries, but in Egypt and Indonesia the virus seems to have become endemic [7,11-13]. In some of these endemically infected countries vaccination of breeders and layer hens has become a widely used containment strategy that has met with variable success [14-17]. In Indonesia, vaccination is widely applied, but it is unclear what the epidemiological situation is, as no official data are available for commercial flocks [18]. Despite vaccination, it is believed that outbreaks in commercial flocks continue to occur. An indication for this is that on several poultry collecting facility houses in Jakarta, where spent layers and broilers were collected shortly before slaughtering, H5 virus was isolated [8,19]. Another indication is the observation of an H5N1 outbreak in a vaccinated commercial layer flock [20].

The situation has not improved in Indonesia since the incursion of the virus in 2003 [7,21], and additional control options are urgently needed. Large-scale culling does not seem an option, and, therefore the vaccination strategy in endemically infected countries needs improvement. A large part of the poultry industry in Indonesia consists of broiler flocks, which are generally not vaccinated. The main reasons for the non-vaccination strategy are the costs of vaccination, and the assumed ineffectiveness of vaccination of broilers because of the interference of maternally derived antibodies (MDA) with a vaccine-induced immune response [17].

To be able to make an informed decision on whether or not vaccination of broilers is useful in the control of HPAI H5N1, and to investigate whether broilers were able to transmit the infection at all, we investigated the effect of vaccination on virus transmission. Focus was on key epidemiological parameters such as the transmission rate parameter, the infectious period, the generation interval, and the reproduction number [22-24]. Estimation of these parameters in the field is possible, but difficult even in endemically infected areas [20]. Experimental transmission studies offer the opportunity to quantify these parameters under well-controlled conditions [4,25]. Here we present the results of four sets of experiments with HPAI H5N1 virus strain A/Chicken/Legok/2003 in broiler chickens, to estimate epidemiological parameters, and to determine the effect of a single vaccination on transmission and clinical signs.

## Materials and methods

Experiments were carried out in the high containment unit at PT. Medion, Bandung, Indonesia. Four experiments were done each with 22 replicates. Each replicate consisted of one pair of broilers. Experiment 1 was carried out with unvaccinated birds, experiment 2 with birds vaccinated at day 1 of age, and experiment 3 with birds vaccinated at day 10. Experiment 4 consisted of progeny obtained from the same flock, but birds were day-old at time of challenge (see below).

### Animals and housing

Approximately 200 18-day-old embryonated eggs were purchased from a commercial breeder farm. The breeders were vaccinated several times against H5N1 with Medivac^® ^(PT Medion, Bandung, Indonesia), containing H5N1 virus strain A/chicken/Legok/2003. At time of purchase of the eggs, no clinical signs of AI in the breeders were reported, indicating the absence of HPAI H5N1 virus at that time.

After hatching at the facilities, day-old chicks were housed in one experimental unit. They were fed with a commercial ration, and had tap water ad libitum. Four groups were formed each consisting of 44 birds for each experiment one. Three groups (experiments 1-3) were of the same age at purchase and challenge and were used to determine the efficacy of vaccination. A fourth group (experiment 4) consisted of day-old chicks (DOC), and were challenged to determine whether virus could be transmitted amongst DOC. Challenge was done when birds in experiments 1-3 were 28 days old.

One week before challenge birds from experiments 1-3 were moved to the experimental units. Two rooms were available, each with two rows with cages on three levels. Birds in experiments 2 and 3 were housed together in Unit 1 and birds in experiment 1 were housed together with those in experiment 4 in Unit 2: each experimental group at opposite sides of the corridor in each house. In each cage one pair of birds from the same experiment was housed. The cages between each experimental pair were empty. Sentinel birds were placed in empty cages in the middle level below each pair in the upper level. Sentinels were SPF layers, from the SPF unit of PT Medion, and were not older than the experimental birds. Sentinels were used to monitor between-cage virus transmission. The experiments lasted four weeks after inoculation, when the surviving birds in experiments 1-3 were 56 days old.

### Vaccine

An inactivated oil-emulsion vaccine was used, which contained the H5N1 strain A/Chicken/Legok/2003 (Medivac^®^, PT Medion, Bandung, Indonesia) [26]. The vaccine was administrated intramuscularly in one leg using 0.5 mL containing 256 HAU per dose per bird. Chickens in experiments 1 and DOC in experiment 4 remained unvaccinated; chickens in experiment 2 were vaccinated at one day of age; the birds in experiment 3 were vaccinated at 10 days of age.

### Inoculation

The HPAI virus strain H5N1 A/Chicken/Legok/2003 was used for challenge. The strain was provided by PT. Medion Bandung Indonesia. The virus has been used in other transmission experiments, and was able to induce clinical signs and transmission [26,27]. At day of challenge, when the birds in experiments 1-3 were 28 days old and in the DOC experiment (4) were 1 day old, one bird per pair was inoculated intranasally and intratracheally with 0.2 mL inoculum containing 10^6^/mL median egg infectious dose (EID_50_) [26]. Before inoculation each bird that had to be inoculated was put in the empty cage near its pen mate. Eight hours after inoculation, they were placed back in their original cage.

### Transmission experiments

Throughout, we refer to each experimental pair of chickens as a trial. Each experiment consisted of 22 replicate trials, and in each trial one inoculated bird (*I*) was placed in a cage with an uninfected contact bird (*C*). Both birds had received the same treatment, and were of the same age.

Transmission of virus was monitored by taking daily swab samples from the trachea and cloaca from all birds for 10 days. From birds that survived this sampling period, additional samples were taken at day 14 after challenge. The samples were stored at -70 °C until further testing. Serum blood samples were taken from surplus birds at day of hatch to determine the level of maternally derived antibodies. From the experimental birds, serum blood samples were taken two days before challenge and four weeks after challenge, at the end of the experiment. Sera were stored at -20°C until further testing. Clinical signs were recorded during four weeks after challenge.

**Figure 1:**
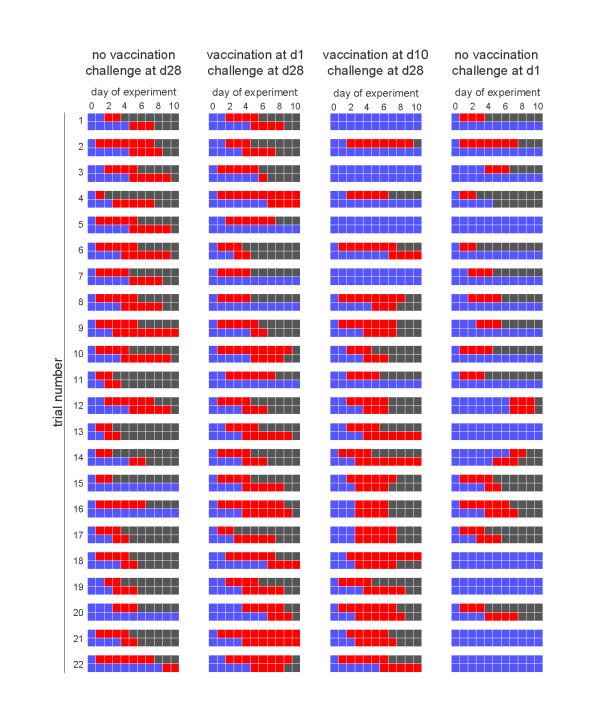
**Overview of the transmission studies**. Shown are for each of the four experiments the experimental data of the 22 replicate trials. The top and bottom rows of each trial refer to the experimentally infected chicken and contact chicken, respectively. Blue squares denote chickens that tested negative, red squares represent chickens that tested positive, and black squares denote chickens that died or recovered after infection.

The treatment is referred to as: unvaccinated (experiment 1), d1 vaccinated (experiment 2), d10 vaccinated (experiment 3) and DOC (experiment 4). Figure 1 gives an overview of the experimental data from days 0 to 10 after challenge. Additional samples were taken from infectious birds that were still present at day 10 of the experiment (not shown). The complete dataset is available on request from the corresponding author. All experiments were carried out in accordance to article 80 on "Research in Animal Health" of the Indonesian "Law on Livestock and Animal Health UU/18/2009".

### Tests

The presence of AI virus in swabs was determined by virus isolation according to standard procedure described by OIE [28]. Briefly, three SPF embryonated chicken eggs, incubated for nine days, were inoculated with 0.2 mL swab medium per egg. After 72 h, or when the embryo had died before that time, the allantoic fluid was harvested. A haemagglutination assay (HA) was performed following standard procedure. When at least one of the eggs was positive in the HA, the swab was considered to be positive. The test results were recorded as positive for AI virus or negative [26]. A bird was considered infected if at least one sample (either tracheal or cloacal) tested positive at least once.

Serum samples were tested in a haemagglutination inhibition (HI) test according to standard procedures [28]. It is generally assumed that HI titers ≥ 32 are protective against disease, i.e. clinical signs [29]. Tests were carried out in duplo using 4 HAU of the strain A/chicken/Legok/2003(H5N1). Two-fold dilutions of the serum samples were made, and titers were expressed as the serum dilution that caused complete inhibition of agglutination [28].

### Quantification of transmission

A Bayesian inferential framework based on a stochastic SEIR (susceptible-exposed-infected and infectious-removed) epidemic model was used to obtain quantitative estimates of the parameters of interest [26,30-32]. The methods of analysis have been described in detail earlier [26]. Here we give a concise overview.

The main interest is in the transmission rate parameter, and parameters of the latent and infectious period distributions. Together, these parameters determine the basic reproduction number and the generation interval [26]. We parameterize the latent and infectious periods using gamma distributions, and assume uninformative uniform prior distributions (*U(0.0001-100)*) for all parameters. To be precise, we characterize gamma distributions of the latent and infectious periods by their mean and variance (and not the shape and scale parameters), and assume uniform prior distributions for the mean and variance (and not the shape and scale parameters).

In the following *β *denotes the transmission rate parameter, *γ_E _*and *δ_E _*the parameters determining the latent period probability distribution, and *γ_I _*and *δ_I _*the parameters of the infectious period probability distribution. Specifically, *E*[*T_E_*] = γ*_E _*and, *Var*[*T_E_*] = *δ_E_*, and *E*[*T_I_*] = γ*_I _*and *Var*[*T_I_*] = *δ_I _*represent the means and variances of these distributions. The corresponding probability densities are denoted by *f_E _*(*x*) and *f_I _*(*x*).

Further, **e**_*k *_**i***_k_*, and **r***_k _*are *N-*dimensional vectors which contain the time points of the S→E, E→I, and I→R transitions for inoculated (*k *= 1) and contact (*k *= 2) birds in the *N *trials. Hence, we have **e**_1 _= (0,...,0)*^T ^*by definition, and all other transition times are unknown. The unknown transitions are imputed. We adopt the convention that *e*_2*j *_denotes the exact time at which the contact bird in experiment *j *is infected, that *i*_1*j *_denotes the exact time that the inoculated bird in experiment *j *became infectious, etc.

As in [26], the contribution of trial *j *to the likelihood is given by(1)

In the above equation *λ*^*(j)*^(*t*) and *S*^*(j)*^(*t*) denote the infection hazard in trial *j *at time *t *and the probability that the contact bird in trial *j *remains uninfected up to time *t*, respectively. If we let [...] denote the indicator function, the infection hazard is given by(2)

where the parameter *t*_*add *_represents the delay between the moment of inoculation and the moment that the inoculated birds were placed back in their cages. Hence, the function max(*t*_*add*_,*i*_1*j*_) marks the beginning of the at-risk period for the contact bird. In all trials and experiments, the delay is 8 h, i.e. *t*_*add *_= 0.33 (*day*). The probability that the contact bird in trial *j *remains uninfected up to time *t *can be expressed in terms of the infection hazard as follows(3)

With the above preparation at hand, the likelihood function is given by the product of the contributions of the individual trials given in equation 1. The above equations are furthermore readily generalized to include differences in the epidemiological parameters of inoculated versus contact birds [26].

The epidemiological parameters and unobserved epidemiological transitions (i.e. S→E, E→I, I→R) were all updated by a random-walk Metropolis algorithm. We used Normal proposal distributions with the current value as mean. After running a number of analyses to explore the posterior distribution and optimize the proposal distributions, we used standard deviations of 0.02 for the epidemiological transitions, and 0.02-0.5 for the epidemiological parameters. The epidemiological parameters and unobserved transitions were updated in blocks, in the order (1) timing of inoculated chickens becoming infectious, (2) timing of removal of inoculated chickens, (3) timing of infection of contact chickens, (4) timing of contact chickens becoming infectious, (5) timing of removal of contact chickens, and (6) updating of the epidemiological parameters [26]. Chains were run for 350 000 cycles, of which the first 100 000 cycles were discarded as burn-in. Thinning was applied by taking output from each twentieth cycle, yielding a sample of 12 500.

Below we report not only the basic epidemiological parameters (transmissibility, duration of the latent and infectious periods), but also the generation interval and basic reproduction number. The generation interval is defined as the moment of infection of the contact bird relative to the moment of infection of the inoculated bird (i.e. it is given by *e*_2*j *_in trial *j *if the contact bird was infected), while the basic reproduction number is defined as the product of the transmission rate parameter (unit: *day^-1^*) and infectious period (unit: *day*).

Each of the Experiments 1-4 was analysed separately, assuming a common distribution of the latent period of inoculated and contact birds. Based on the results of the separate experiments, and given the observation that there may be differences between inoculated and contact birds, possibly due to differences in the inoculum size we also analysed the combined data of Experiments 1-3 while relaxing this assumption. Specifically, we allowed the mean of the latent period to differ between inoculated and contact birds, while assuming a fixed common variance (0.001) of the latent periods [26]. Furthermore, the data of Experiments 1-3 were used to explore, by means of logistic regression, whether the probability of infection could be dependent on the immune status (i.e. HI titer) of the birds just prior to the experiments.

## Results

### Experiment 1 (no vaccination, challenge at day 28)

All inoculated chickens shed virus for 1-7 days (interquartile range: 2-5 days), and all inoculated chickens died 2-8 days post challenge (interquartile range: 3-6 days post challenge) (Table 1, Figure 1). Likewise, all but two of the contact chickens shed virus for 2 to more than 8 days. All virus-positive inoculated chickens showed clinical signs of highly pathogenic avian influenza (data not shown) and died, and 16 of 22 contact chickens died during the course of the experiments. At challenge none of the birds had a HI titer greater than or equal to 32, and only 1 of the surviving contact chickens had developed a significant HI titer (≥ 32) at the end of the experiment. The average HI titers are represented in Table 1.

**Table 1:** Overview of HI titers at challenge, virus isolation data, clinical symptoms, and mortality rates

				Number of birds							
		
		Mean HI titer (absolute (sd))		**with HI titer **≥ 2^5 at challenge		shedding virus^a^		with clincial symptoms		that died	
**Exp.**	**Treatment**	**at challenge** **(**^**2**^**log)**	**at end**^**d **^ **(**^**2**^**log)**	**I**^**b**^	**C**^**b**^	**I**	**C**	**I**	**C**	**I**	**C**

1	no vaccination challenge at d28 of age	1.6 (1.0)	206 (457)	0	0	22	19	22	22	22	16
2	vaccination at d1 challenge at d28 of age	1.7 (1.3)	166 (381)	0	0	22	18	19	16	16	15
3	vaccination at d10 challenge at d28 of age	1.8 (0.9)	251 (357)	0	0	18	15	16	10	13	7
4	no vaccination challenge at day 1 of age	4.9 (1.2)^c^	1.0 (0.4)^f^	Nd^c^	nd	16	6	19	19	13	1

^a ^total numbers in each group were 22 inoculated chickens and 22 contact-exposed chickens.

^b ^I: inoculated chickens; C: contact-exposed chickens.

^c ^not determined.

^d ^only from surviving and infected birds.

^e ^from surplus birds.

^f ^one bird had a titer of 1024, which is omitted from this average.

**Figure 2:**
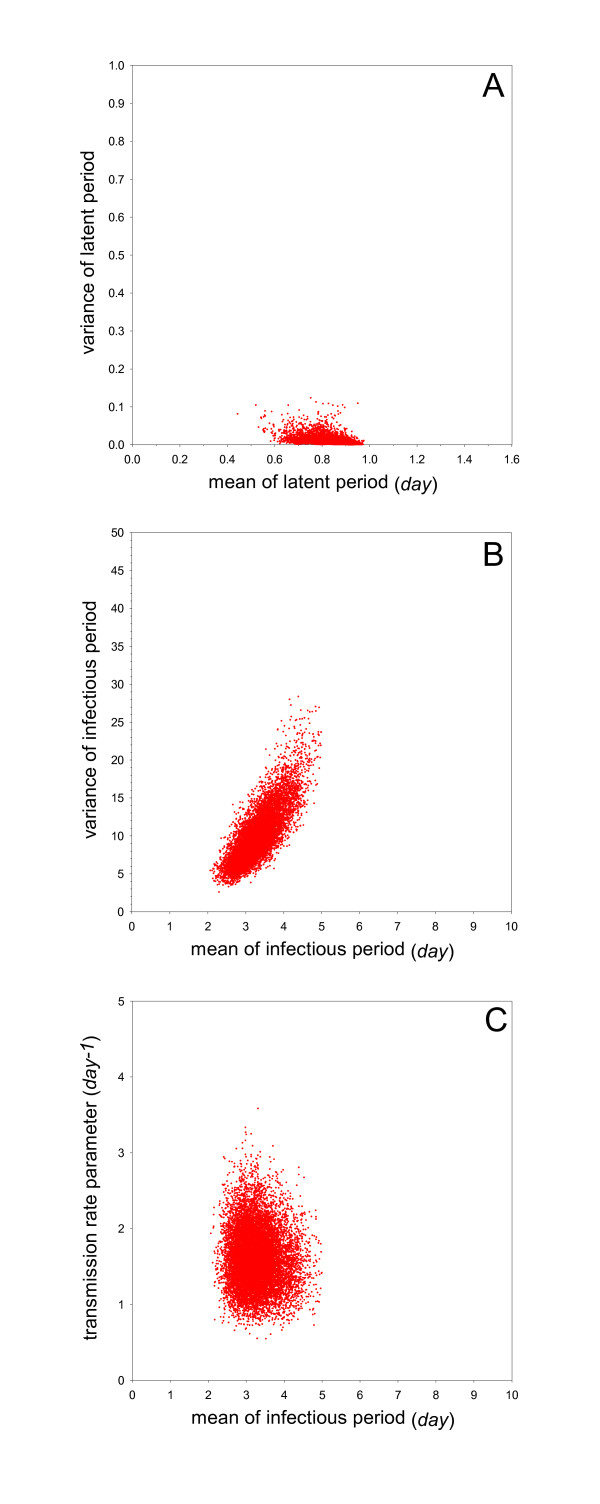
**Overview of the analyses of Experiment 1 (no vaccination, challenge at day 28)**. Shown are samples from the marginal posterior density of the mean versus variance of the latent period (A), the mean versus variance of the infectious period (B), and the mean infectious period versus transmission rate parameter (C).

The estimated transmission rate parameter (i.e. the median of the posterior distribution of the transmission rate parameter) is 1.6 per day (95%CrI: 0.97-2.4) (Table 2). This implies that the estimated per day probability of infection of an uninfected contact chicken by an infected inoculated chicken is 1 - exp( - 1.6) = 0.80. The estimated mean of the infectious period (i.e. the median of the posterior distribution of the parameter determining the mean of the infectious period) is 3.2 days (95%CrI: 2.5-4.3), while the estimated mean of the latent period is 0.88 days (95%CrI: 0.70-0.94) (Table 2). Based on these estimates, the basic reproduction number and the generation interval are 5.1 (95%CrI: 3.0-8.4) and 1.5 days (95%CrI: 1.3-1.7), respectively. Figure 2 gives a graphical representation of the posterior distribution of the mean versus variance of the latent period, and of the infectious period and the mean infectious period versus the transmission rate parameter.

**Table 2:** Overview of the statistical analyses

**Exp**.	Treatment	Transmission parameter (*day^-1^*)(95%CrI)	Latent period (*day*)(95%CrI)	Infectious period (*day*)(95%CrI)	Reproduction number (95%CrI)	Generation interval (*day*)(95%CrI)
1	no vaccination challenge at day 28	1.6 (0.97-2.4)	0.88 (0.70-0.94)	3.2 (2.5-4.3)	5.1 (3.0-8.4)	1.5 (1.3-1.7)
2	vaccination at day 1 challenge at day 28	1.5 (0.87-2.3)	0.86 (0.69-0.96)	3.7 (2.8-5.1)	5.5 (3.1-9.3)	1.4 (1.2-1.5)
3	vaccination at day 10 challenge at day 28	1.3 (0.69-2.1)	0.89 (0.56-1.1)	3.5 (2.5-5.2)	4.4 (2.3-8.3)	1.4 (1.2-1.5)
4	no vaccination challenge at day 1	0.38 (0.17-0.72)	3.3 (2.4-4.1)	2.8 (2.1-3.7)	1.0 (0.45-2.1)	1.1 (0.77-1.3)
1-3	challenge at day 28	1.4 (1.1-1.9)	i: 0.93 (0.88-0.96) c: 0.96 (0.85-1.1)	3.3 (2.7-3.9)	4.6 (3.3-6.4)	1.4 (1.3-1.5)

Parameter estimates are given as posterior medians. Equal-tailed 95% credible intervals are shown between brackets. Estimates of the latent period in the combined analysis of Experiments 1-3 refer to the inoculated (i) and contact (c) birds.

### Experiment 2 (vaccination at day 1, challenge at day 28)

All inoculated chickens shed virus, and 16 of 22 inoculated chickens died before the end of the experiment. Seventeen contact chickens shed virus, and 14 of 22 contact chickens died during the course of the experiments. At challenge none of the birds had a HI titer greater than or equal to 32; 4 of the surviving inoculated and none of the surviving contact chickens developed a significant HI titer (≥ 32) at the end of the experiment. The average HI titers are represented in Table 1.

**Figure 3:**
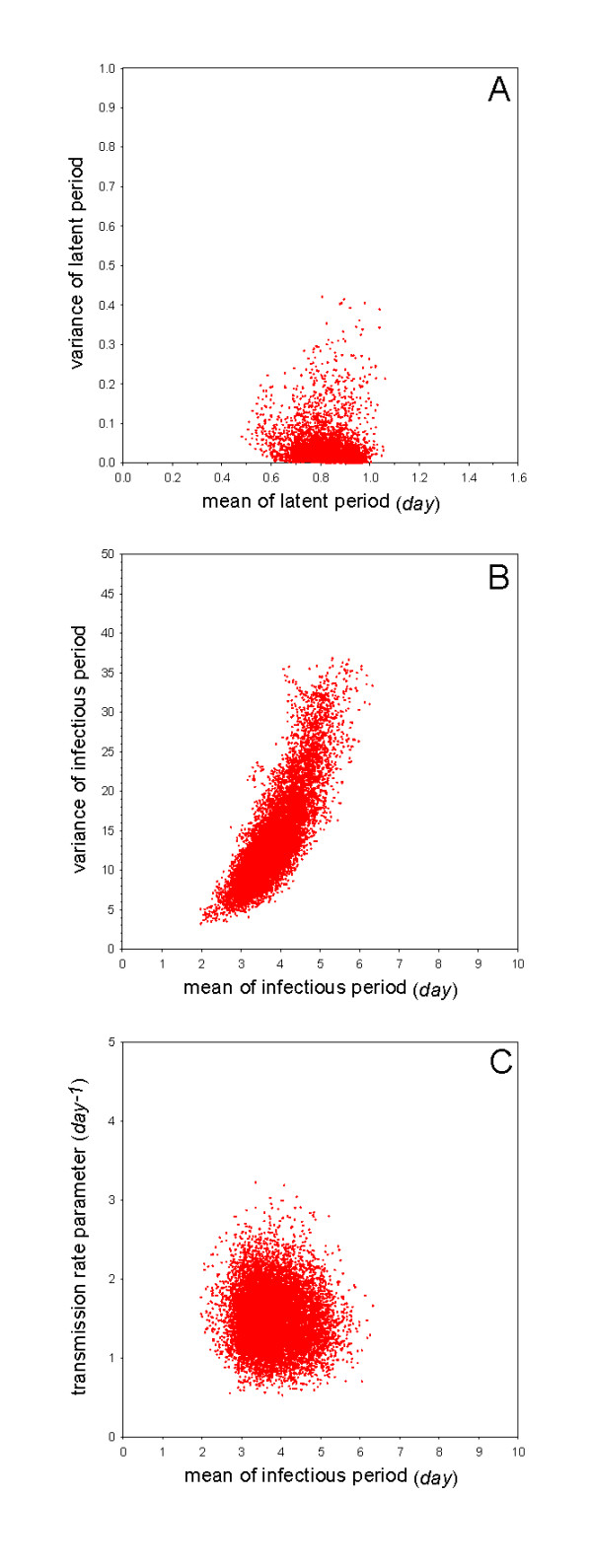
**Overview of the analyses of Experiment 2 (vaccination at day 1, challenge at day 28)**. See Figure 1 for details.

The basic parameters of interest are very close to those of experiment 1 (no vaccination). Specifically, the estimated transmission parameter is 1.5 per day (95%CrI: 0.87-2.3), the mean of the latent period is 0.86 days (95%CrI: 0.69-0.96), and the mean of the infectious period is 3.7 days (95%CrI: 2.8-5.1) (Table 2). The basic reproduction number and generation interval are also quite close to the estimates in experiment 1, *viz*. 5.5 (95%CrI: 3.1-9.3) for the reproduction number, and 1.4 days (95%CrI: 1.2-1.5) for the generation interval (Table 2). Figure 3 shows that although there is a striking overall agreement with the results of experiment 1 (Figure 2), there is also some evidence of greater variability in experiment 2 than in experiment 1, especially with respect to the variance of the latent and infectious periods.

### Experiment 3 (vaccination at day 10, challenge at day 28)

Eighteen inoculated chickens shed virus, and 13 died of AI (Table 2 Figure 3). Likewise, 15 contact chickens shed virus and 15 of 22 contact chickens survived, including the 7 that did not shed detectable levels of virus. At challenge none of the birds had a HI titer greater than or equal to 32; 5 of the surviving inoculated and 8 of the contact chickens developed a significant HI titer (≥ 32) at the end of the experiment. The average HI titers are represented in Table 1. Three contact chickens and the inoculated one that did not develop HI > 32 did not shed virus.

**Figure 4:**
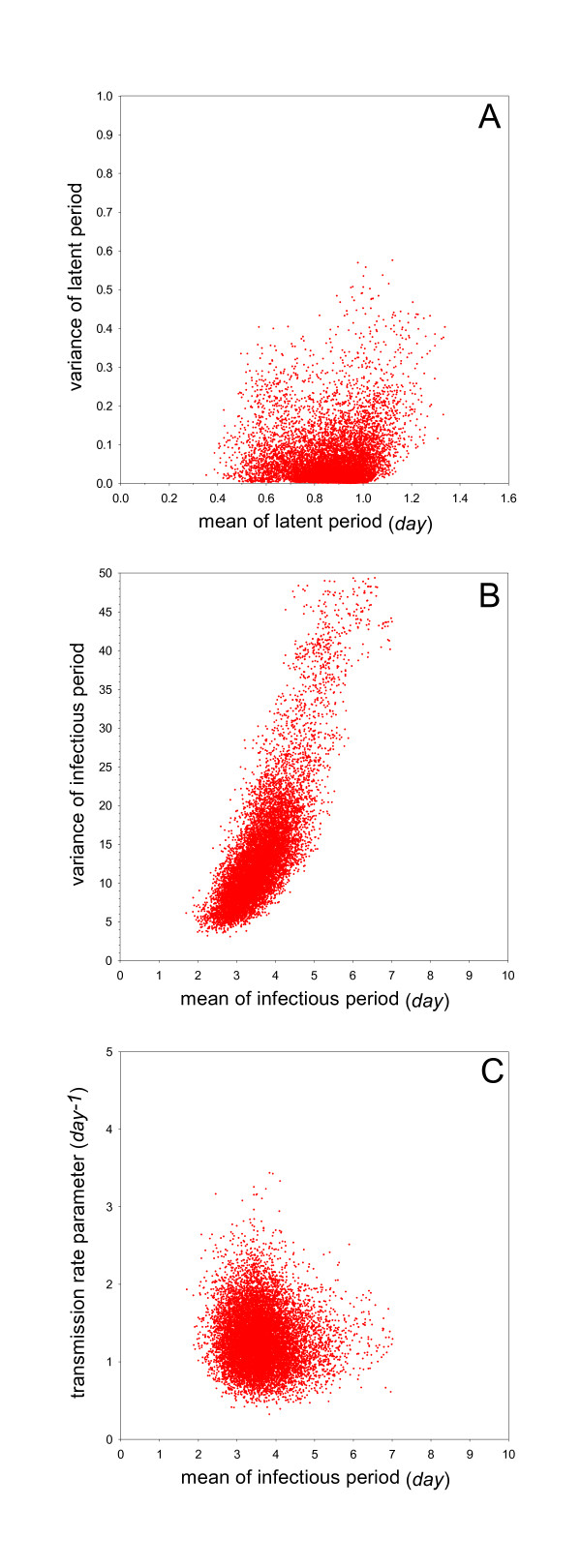
**Overview of the analyses of Experiment 3 (vaccination at day 10, challenge at day 28)**. See Figure 1 for details.

The results of the analysis of experiment 3 (vaccination on the tenth day of life) are also similar to those of experiments 1 (no vaccination) and 2 (vaccination at the first day of life) (Figure 4). Here, estimates of the key parameters are 1.3 per day (95%CrI: 0.69-2.1) for the transmission rate parameter, 0.89 days (95%CrI: 0.56-1.1) for the mean of the latent period, and 3.5 days (95%CrI: 2.5-5.2) for the mean of the infectious period (Table 2). The reproduction number and generation interval are estimated at 4.4 (95%CrI: 2.3-8.3) and 1.4 days (95%CrI: 1.2-1.5), respectively. Figure 4 shows that, in contrast with experiments 1 and 2, the variance of the infectious period cannot be estimated with high precision anymore, indicating that the experimental data contain little information on the variance of the infectious period (Figure 4).

### Experiment 4 (no vaccination, challenge at day 1)

**Figure 5:**
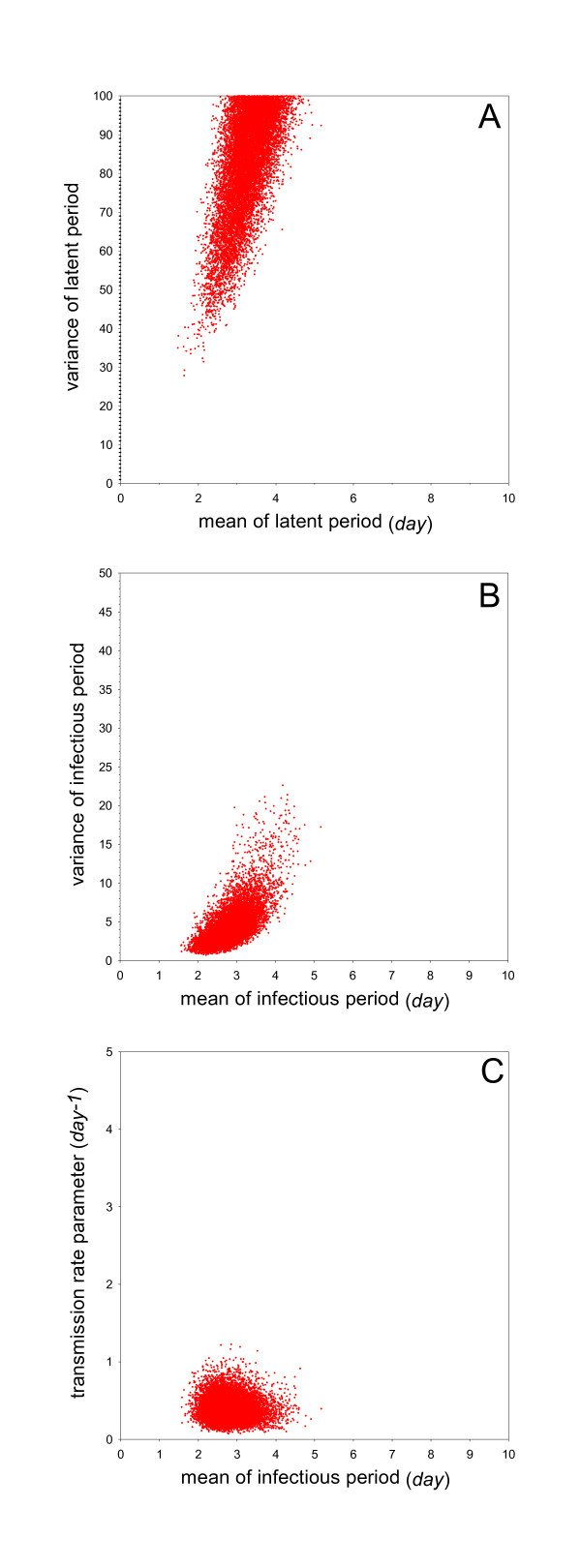
**Overview of the analyses of Experiment 4 (no vaccination, challenge at day 1)**. See Figure 1 for details.

In this experiment, 16 of 22 inoculated birds shed virus, and 13 inoculated birds died (Table 1, Figure 5). Six of the contact birds shed virus, and one died during the course of the experiments. The birds used in the experiment were very young, and could not be tested for the presence of MDA before the experiment. Therefore, eighteen day-old chickens that were not used in the experiments were bled, and tested for the presence of MDA, and 13 of 18 had HI titers ≥32, and had an average HI titer of 4.9.

The analyses of the experiment show substantial differences in the parameter estimates when compared with those resulting from the analyses of experiments 1-3 (challenge at four weeks of age). The median of the posterior of the transmission rate parameter is 0.38 per day (95%CrI: 0.17-0.72), and the medians of the posterior distribution of the mean of the latent and infectious periods are 3.3 days (95%CrI: 2.4-4.1) and 2.8 days (95%CrI: 2.1-3.7), respectively (Table 2). The estimates (i.e. the medians of the posterior distribution) of the reproduction number and generation interval are 1.0 (95%CrI: 0.45-2.1) and 1.1 days (95%CrI: 0.77-1.3), respectively.The data contain little information on the variance of the latent period (Figure 5).

### Combined analysis (vaccination at day 28)

The above analyses indicate that vaccination had no measurable effect on the transmission dynamics when birds were infected and challenged at day 28 (Experiments 1-3; Table 2). We therefore combined and reanalysed the data of these experiments to investigate whether there were differences between inoculated and contact birds in the latent period, and to explore the relation between the HI titer of contact birds prior to the experiment and the probability of infection.

Additional file 1 Figure S1 illustrates that the combined analyses enables more precise estimation of the epidemiological parameters of interest (Table 2). The figure furthermore shows that there are no large differences between the means of the latent period of inoculated versus contact birds.

The result of the logistic regression of infection outcome as a function of HI titer prior to the experiment was largely inconclusive, as only 4 out of 62 contact birds in trials with a successfully infected inoculated bird had not been infected, and as HI titers prior to the experiment were low and showed little variation (four birds had a HI titer of 4, the remainder had HI titers of 0-2). The analysis indicated that the predicted probability of infection decreased from 95% if the initial HI titer was 0, to 93% if the initial HI titer was 2. However, the associated confidence intervals are wide, and the parameter determining the slope is not significantly different from 0 (*p *= 0.63). HI titers of all birds are given in Additional file 2 Table S1.

## Discussion

The aim of this study was to quantify the effect of a single vaccination of broilers against a HPAI H5N1 on virus transmission, and to which extent virus could spread among broilers and DOC. Vaccination with an inactivated homologous strain did not reduce transmission of H5N1 virus significantly. In both vaccine experiments, in which birds were vaccinated when they were either one or ten days old, the reproduction number was above one indicating that virus could still spread extensively. Moreover vaccination did not prevent the occurrence of clinical signs, although it seemed to reduce mortality slightly. This implies that unnoticed virus spread is unlikely, even in a vaccinated flock, which is often feared by farmers and policy makers as mentioned before [14,15,33].

Transmission among day-old chickens, which still had MDA, tended to be reduced compared to chickens of four weeks of age without MDA, as fewer contact birds became infected, but the reproduction number was not significantly below one. Of course the real control group, MDA-free DOC, was lacking, as this was not our research question. In various trials, the clinical signs seemed to be less severe, suggesting that these DOCs may pose a risk in the spread of the infection as they may spread the virus unnoticed. These findings indicate that broilers, including DOC, could play a role in the epidemiology of AI, as virus could spread extensively. It cannot be determined from these experiments, however, to which extent this may occur in the field, as this also depends on the number of virus incursions, and the subsequent implemented control measures.

Vaccination only induced very low titers of HI antibodies in few birds and a single vaccination was not effective in reducing transmission. The most likely explanation for the reduced efficacy in comparison to layers was the presence of maternally derived antibodies at time of vaccination. MDA in general persist in broilers for approximately 14-21 days after hatch [34], and for AI it has been demonstrated that MDA titers were low at 7 days after hatching [35]. It has been demonstrated for other viral infections that MDA may interfere with an effective immune response [36,37], like for example for Newcastle disease [38] and infectious bronchitis [39]. Whether there was interference between MDA and vaccination could not be demonstrated in this study, as no group of broilers without MDA was included, because this type of broilers is not present in endemically infected areas in which broiler breeders are vaccinated regularly, and because it was not the research question of this study.

A study on AI vaccination in MDA-positive broilers [35] demonstrated that HI titers remained high until five weeks post vaccination, indicating that a decrease in immunity was not to be expected within the interval vaccination-challenge applied in our trials. Studies have also shown that HI levels after a single vaccination in birds with MDA reached a peak at six weeks post vaccination [15,35], suggesting our interval being too short. We challenged at 28 days of age, however, as this was assumed to be a reasonable interval for field conditions and also considering the duration of the experiments and applied before [40].

Another explanation for the failing immune response is that broilers have an immature immune system, as broilers have been bred for growth characteristic, which may have an effect on both the humoral and cellular immune responses [41]. Although the birds in our experiments were not protected, other studies have shown that AI vaccination of broilers at 10 days of age gave satisfactory antibody titers and clinical protection after challenge [35,42,43]. A possible explanation for the difference between their observations and ours is the use of different vaccines or adjuvantia.

In our experiment, some birds did survive the infection, although the HI titers at challenge were below 32. In the unvaccinated experiments with 4-week-old birds, for example, all inoculated birds shed virus and showed clinical sign, but some contact-infected chickens survived for more than 10 days. This was also observed in the vaccine experiments and DOC experiment. However, no association could be found between HI titer at moment of challenge and protection against contact-infection and also not between HI titer and infection after inoculation. This phenomenon has also been observed in studies on for example H7N7 in turkeys [44], and on Newcastle disease [45]. In the latter experiment, vaccinated birds with low or undetectable antibody titres were protected against disease and mortality, but infection and transmission still occurred. One explanation for surviving of contact birds is that these birds had become infected with a low virus dose. As the birds were housed in pairs, the exposure dose could have been low, as some of the inoculated chickens in this experiment died before having shed a large amount of virus. However, Spekreijse et al. [46] showed that the case fatality rate of chickens did not differ between dose groups, and in their experiments all birds that were infected died eventually. Another explanation is that other immune responses than antibodies were induced, such as a cellular immunity, although it is generally assumed that inactivated vaccines usually induce a B cell response only [47]. Another possibility is that the low HI titers may have been sufficiently high to induce some protection.

Of course extrapolation of results from experiments to the field should always be done with caution. Nevertheless, our results indicate that vaccination is not effective in broilers, as early vaccination does not induce a good immune response and if vaccination is applied later, the birds may be protected the moment they are slaughtered. Henning et al. [48] demonstrated that the risk of infection was higher in flocks vaccinated once, in comparison to two vaccinations, also suggesting that vaccination of broilers will not be very effective, but it does not seem to be feasible vaccinating broilers even twice. Although discrepancies between laboratory and field results have been observed more often [49], the efficacy of vaccination is usually higher under experimental conditions than under field conditions. Therefore, a proper vaccination scheme with killed vaccines seems useless, although use of other types of vaccines [17,33] could be more successful. Adequate biosecurity measures should therefore be implemented in endemically infected countries to control AI [2,14,15,17,50].

## Competing interests

The authors declare that they have no competing interests.

## Authors' contributions

OP is a PhD student, and was involved in the animal experiment, statistical analyses, and drafter of the manuscript. AB was the daily supervisor, involved in design of the trials, assisting in the writing process. IC participated in the design and organisation of the trials. GK participated in the design and organisation of the trials. RS was the Indonesian supervisor, and was involved in design of the trials. AS is the promotor, and participated in discussions about the design; MB carried out statistical analyses and mathematical modeling. All authors read and approved the final manuscript.

## Acknowledgements

This research was carried out as part of the Indonesian-Dutch bilateral program on the control of HPAI in Indonesia, which is funded by the Dutch Ministry of Agriculture, Nature and Food Safety (The Hague, The Netherlands).

## Supplementary Material

Additional file 1 **Figure S1. Overview of the combined analyses of Experiments 1-3 (challenge at day 28)**. The top panel shows the marginal posterior distribution of the mean of the latent period of the inoculated versus contact birds. See Figure 2 for further details. Click here for file

Additional file 2 **Table S1. HI titers at time of challenge and at the end of the trial (4 weeks after inoculation)**. Titers are expressed as 2 fold dilution (titers of DOC and of other birds at hatch or at time of vaccination could not be determined, because the birds were very small and we did not want to take a risk that birds might die due to blood collection. Click here for file
