# Supplementary figures and images for: A single vaccination of commercial broilers does not reduce transmission of H5N1 highly pathogenic avian influenza
Source: Vet Res. 2011 Jun 2;42(1):74. doi: 10.1186/1297-9716-42-74 (PMC3132710; doi:10.1186/1297-9716-42-74)

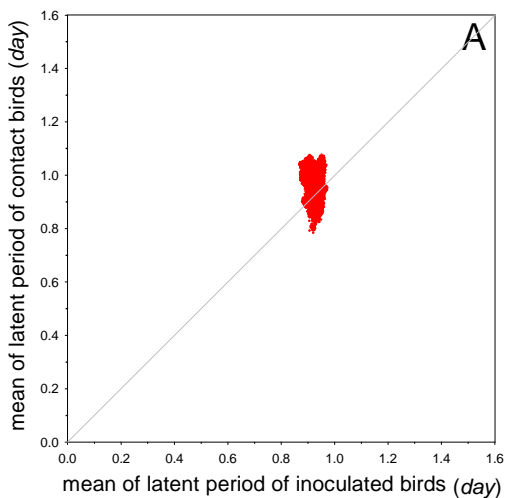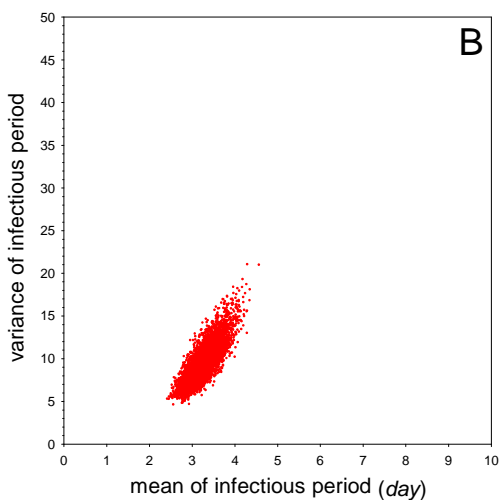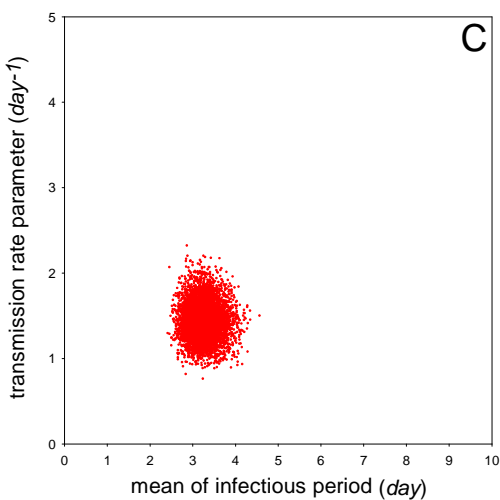

Supplement: Additional file 1 — Figure S1. Overview of the combined analyses of Experiments 1-3 (challenge at day 28). The top panel shows the marginal posterior distribution of the mean of the latent period of the inoculated versus contact birds. See Figure 2 for further details. [file 1297-9716-42-74-S1.PDF]
