# Supplementary material for: A single vaccination of commercial broilers does not reduce transmission of H5N1 highly pathogenic avian influenza
Source: Vet Res. 2011 Jun 2;42(1):74. doi: 10.1186/1297-9716-42-74 (PMC3132710; doi:10.1186/1297-9716-42-74)
Supplement: Additional file 2 — Table S1. HI titers at time of challenge and at the end of the trial (4 weeks after inoculation). Titers are expressed as 2 fold dilution (titers of DOC and of other birds at hatch or at time of vaccination could not be determined, because the birds were very small and we did not want to take a risk that birds might die due to blood collection. [file 1297-9716-42-74-S2.DOC]

Additional file

Table S1: HI titers at time of challenge and at the end of the trial (4 weeks after inoculation). Titers are expressed as 2 fold dilution (titers of DOC and of other birds at hatch or at time of vaccination could not be determined, because the birds were very small and we did not want to take a risk that birds might die due to blood collection.

| Treatment group | Pair no. | Inoculated (i) /contact (c) | HI titer at challenge^a^ | HI titer at end of experiment^b^ | Infected ^c^ |
| --- | --- | --- | --- | --- | --- |
| Unvaccinated | 1 | I | 1 | * | 1 |
|  |  | C | 1 | * | 1 |
|  | 2 | I | 2 | * | 1 |
|  |  | C | 2 | * | 1 |
|  | 3 | I | 2 | * | 1 |
|  |  | C | 2 | * | 1 |
|  | 4 | I | 2 | * | 1 |
|  |  | c | 2 | * | 1 |
|  | 5 | i | 2 | * | 1 |
|  |  | c | 2 | * | 1 |
|  | 6 | i | 1 | * | 1 |
|  |  | c | 1 | * | 1 |
|  | 7 | i | 1 | * | 1 |
|  |  | c | 1 | * | 1 |
|  | 8 | i | 1 | 1 | 1 |
|  |  | c | 1 | * | 1 |
|  | 9 | i | 1 | * | 1 |
|  |  | c | 1 | * | 1 |
|  | 10 | i | 1 | * | 1 |
|  |  | c | 1 | * | 1 |
|  | 11 | i | 1 | * | 1 |
|  |  | c | 1 | * | 1 |
|  | 12 | i | 4 | * | 1 |
|  |  | c | 2 | 1024 | 1 |
|  | 13 | i | 1 | * | 1 |
|  |  | c | 1 | * | 1 |
|  | 14 | i | 2 | * | 1 |
|  |  | c | 2 | * | 1 |
|  | 15 | i | 1 | * | 1 |
|  |  | c | 2 | * | 1 |
|  | 16 | i | 1 | * | 1 |
|  |  | c | 4 | * | 1 |
|  | 17 | i | 4 | * | 1 |
|  |  | c | 1 | 1 | 1 |
|  | 18 | i | 1 | * | 1 |
|  |  | c | 1 | 1 | 1 |
|  | 19 | i | 4 | * | 1 |
|  |  | c | 1 | 1 | 1 |
|  | 20 | i | 1 | * | 1 |
|  |  | c | 1 | * | 1 |
|  | 21 | i | 1 | * | 1 |
|  |  | c | 1 | 1 | 1 |
|  | 22 | i | 2 | * | 1 |
|  |  | c | 4 | * | 1 |
|  |  |  |  |  |  |
| Vaccinated at day 1 | 1 | i | 1 | 1 | 1 |
|  |  | c | 1 | * | 1 |
|  | 2 | i | 1 | * | 1 |
|  |  | c | 1 | * | 1 |
|  | 3 | i | 2 | * | 1 |
|  |  | c | 1 | * | 1 |
|  | 4 | i | 2 | * | 1 |
|  |  | c | 2 | * | 1 |
|  | 5 | i | 4 | 64 | 1 |
|  |  | c | 2 | 4 | 0 |
|  | 6 | i | 1 | * | 1 |
|  |  | c | 1 | * | 1 |
|  | 7 | i | 1 | * | 1 |
|  |  | c | 1 | 2 | 1 |
|  | 8 | i | 1 | * | 1 |
|  |  | c | 1 | 1 | 0 |
|  | 9 | i | 1 | * | 1 |
|  |  | c | 1 | * | 1 |
|  | 10 | i | 1 | 1024 | 1 |
|  |  | c | 1 | * | 1 |
|  | 11 | i | 1 | 32 | 1 |
|  |  | c | 1 | 1 | 1 |
|  | 12 | i | 2 | * | 1 |
|  |  | c | 0 | * | 1 |
|  | 13 | i | 1 | * | 1 |
|  |  | c | 4 | 1 | 1 |
|  | 14 | i | 1 | * | 1 |
|  |  | c | 1 | * | 1 |
|  | 15 | i | 1 | * | 1 |
|  |  | c | 2 | * | 1 |
|  | 16 | i | 8 | 1024 | 1 |
|  |  | c | 2 | * | 1 |
|  | 17 | i | 1 | * | 1 |
|  |  | c | 1 | * | 1 |
|  | 18 | i | 4 | 8 | 1 |
|  |  | c | 1 | 1 | 1 |
|  | 19 | i | 2 | * | 1 |
|  |  | c | 1 | * | 1 |
|  | 20 | i | 2 | 2 | 1 |
|  |  | c | 2 | 1 | 1 |
|  | 21 | i | 2 | * | 1 |
|  |  | c | 2 | * | 1 |
|  | 22 | i | 2 | * | 1 |
|  |  | c | 2 | 2 | 1 |
|  |  |  |  |  |  |
| Vaccinated at day 10 | 1 | i | 2 | 128 | 0 |
|  |  | c | 2 | 2 | 0 |
|  | 2 | i | 2 | * | 1 |
|  |  | c | 2 | 128 | 0 |
|  | 3 | i | 2 | 32 | 0 |
|  |  | c | 2 | 1 | 0 |
|  | 4 | i | 2 | * | 1 |
|  |  | c | 2 | 32 | 1 |
|  | 5 | i | 4 | 16 | 0 |
|  |  | c | 2 | 4 | 0 |
|  | 6 | i | 1 | 256 | 1 |
|  |  | c | 1 | * | 1 |
|  | 7 | i | 1 | 16 | 0 |
|  |  | c | 1 | 2 | 0 |
|  | 8 | i | 1 | * | 1 |
|  |  | c | 1 | * | 1 |
|  | 9 | i | 1 | * | 1 |
|  |  | c | 1 | 512 | 1 |
|  | 10 | i | 1 | * | 1 |
|  |  | c | 1 | * | 1 |
|  | 11 | i | 2 | * | 1 |
|  |  | c | 2 | 128 | 0 |
|  | 12 | i | 4 | * | 1 |
|  |  | c | 1 | 256 | 1 |
|  | 13 | i | 1 | * | 1 |
|  |  | c | 2 | * | 1 |
|  | 14 | i | 1 | * | 1 |
|  |  | c | 1 | 1024 | 1 |
|  | 15 | i | 1 | * | 1 |
|  |  | c | 4 | * | 1 |
|  | 16 | i | 4 | 64 | 1 |
|  |  | c | 2 | 4 | 1 |
|  | 17 | i | 1 | 0 | 1 |
|  |  | c | 2 | 0 | 1 |
|  | 18 | i | 4 | 128 | 1 |
|  |  | c | 2 | 128 | 1 |
|  | 19 | i | 1 | * | 1 |
|  |  | c | 1 | * | 1 |
|  | 20 | i | 2 | * | 1 |
|  |  | c | 1 | * | 1 |
|  | 21 | i | 2 | 64 | 1 |
|  |  | c | 2 | 16 | 1 |
|  | 22 | i | 1 | * | 1 |
|  |  | c | 1 | 1024 | 1 |
|  |  |  |  |  |  |
| DOC | 1 | i | nd | * | 1 |
|  |  | c | nd | 1 | 0 |
|  | 2 | i | nd | * | 1 |
|  |  | c | nd | 1 | 0 |
|  | 3 | i | nd | * | 1 |
|  |  | c | nd | 1 | 0 |
|  | 4 | i | nd | * | 1 |
|  |  | c | nd | * | 1 |
|  | 5 | i | nd | 1 | 1 |
|  |  | c | nd | 1 | 0 |
|  | 6 | i | nd | * | 1 |
|  |  | c | nd | 1 | 0 |
|  | 7 | i | nd | * | 1 |
|  |  | c | nd | 1 | 0 |
|  | 8 | i | nd | * | 1 |
|  |  | c | nd | 1 | 0 |
|  | 9 | i | nd | * | 1 |
|  |  | c | nd | * | 1 |
|  | 10 | i | nd | * | 1 |
|  |  | c | nd | 1 | 0 |
|  | 11 | i | nd | * | 1 |
|  |  | c | nd | 1 | 0 |
|  | 12 | i | nd | * | 1 |
|  |  | c | nd | 1 | 1 |
|  | 13 | i | nd | 2 | 1 |
|  |  | c | nd | 1 | 1 |
|  | 14 | i | nd | 1 | 1 |
|  |  | c | nd | 1 | 1 |
|  | 15 | i | nd | 1 | 1 |
|  |  | c | nd | 1 | 1 |
|  | 16 | i | nd | 1024 | 1 |
|  |  | c | nd | 1 | 1 |
|  | 17 | i | nd | * | 1 |
|  |  | c | nd | 1 | 1 |
|  | 18 | i | nd | 1 | 0 |
|  |  | c | nd | 1 | 0 |
|  | 19 | i | nd | 1 | 0 |
|  |  | c | nd | 1 | 0 |
|  | 20 | i | nd | * |  |
|  |  | c | nd | 1 | 1 |
|  | 21 | i | nd | 1 | 0 |
|  |  | c | nd | 1 | 0 |
|  | 22 | i | nd | 0 | 0 |
|  |  | c | nd | 1 | 0 |

^a^ expressed are the two-fold dilution

^b^ * the bird died before the end of the trial and the titer could not be determined

^c^ 1 means that the bird was infected

nd not determined
